# The status of machine learning in HIV testing in South Africa: a qualitative inquiry with stakeholders in Gauteng province

**DOI:** 10.3389/fdgth.2025.1618781

**Published:** 2025-08-01

**Authors:** Musa Jaiteh, Edith Phalane, Yegnanew A. Shiferaw, Refilwe Nancy Phaswana-Mafuya

**Affiliations:** ^1^ South African Medical Research Council/University of Johannesburg Pan African Centre for Epidemics Research Extramural Unit, Faculty of Health Sciences, University of Johannesburg, Johannesburg, South Africa; ^2^ Department of Statistics, Faculty of Science, University of Johannesburg, Johannesburg, South Africa

**Keywords:** HIV testing, machine learning, qualitative study, Gauteng province, South Africa

## Abstract

**Background:**

The human immunodeficiency virus (HIV) remains one of the leading causes of death globally, with South Africa bearing a significant burden. As an effective way of reducing HIV transmission, HIV testing interventions are crucial and require the involvement of key stakeholders, including healthcare professionals and policymakers. New technologies like machine learning are remarkably reshaping the healthcare landscape, especially in HIV testing. However, their implementation from the stakeholders’ point of view remains unclear. This study explored the perspectives of key stakeholders in Gauteng Province on the status of machine learning applications in HIV testing in South Africa.

**Methods:**

The study used an exploratory qualitative approach to recruit 15 stakeholders working in government and non-government institutions rendering HIV testing services. The study participants were healthcare professionals such as public health experts, lab scientists, medical doctors, nurses, HIV testing services, and retention counselors. Individual-based in-depth interviews were conducted using open-ended questions. Thematic content analysis was used, and results were presented in themes and sub-themes.

**Results:**

Three main themes were determined, namely awareness level, existing applications, and perceived potential of machine learning in HIV testing interventions. A total of nine sub-themes were discussed in the study: limited knowledge among frontline workers, research vs. implementation gap, need for education, self-testing support, data analysis tools, counseling aids, youth engagement, system efficiency, and data-driven decisions. The study shows that integration of machine learning would enhance HIV risk prediction, individualized testing through HIV self-testing, and youth engagement. This is crucial for reducing HIV transmission, addressing stigma, and optimizing resource allocation. Despite the potential, machine learning is underutilized in HIV testing services beyond statistical analysis in South Africa. Key gaps identified were a lack of implementation of research findings and a lack of awareness among frontline workers and end-users.

**Conclusion:**

Policymakers should design educational programs to improve awareness of existing machine learning initiatives and encourage the implementation of research findings into HIV testing services. A follow-up study should assess the feasibility, structural challenges, and design implementation strategies for the integration of machine learning in HIV testing in South Africa.

## 1 Introduction

The human immunodeficiency virus/acquired immunodeficiency syndrome (HIV/AIDS) remains one of the leading causes of death globally, with South Africa bearing a significant burden (1). With an adult prevalence rate of 15.6%, Gauteng Province ranked sixth in 2022, according to the South African HIV Prevalence, Incidence, and Behavior Survey (SABSSM) (2). As an effective way of reducing HIV transmission, HIV testing interventions are crucial and require the involvement of key stakeholders, including healthcare professionals and policymakers (3, 4). The National HIV Testing Services Policy's emphasis on enhancing HIV Testing Services (HTS) within government and private health facilities significantly improved the uptake and acceptability of HIV testing (4). However, there are still hard-to-reach populations whose HIV status remains unknown, highlighting the need to adopt new testing strategies.

New technologies like artificial intelligence (AI) and machine learning are remarkably reshaping the healthcare landscape, especially in HIV testing (3, 5). Essentially, machine learning algorithms are sets of AI tools used in analyzing complex healthcare data to enhance early diagnosis and treatment of HIV (3, 5–7). Machine learning can be supervised or unsupervised, depending on whether the algorithm is trained on labeled data or the system self-defines the data structure from unlabeled input (8). They are used in various forms. In HIV risk/test predictions, common statistical machine learning tools used include logistic regression, random forest, support vector machines, extreme gradient boosting machines, principal component analysis, etc (5). They can also be useful in enabling HIV self-testing (HIVST) when integrated with digital devices in the form of chatbots or conversational agents (9, 10). Machine learning recently emerged as an integral tool for improving the accuracy and efficacy of HIV diagnostic devices (5).

In establishing prioritized testing and resource optimization, studies have utilized machine learning techniques to predict high-risk individuals, such as key populations (KPs) (5, 8, 11). Such strategies are crucial, especially due to the current reductions in funding for HIV interventions, which heavily affect developing countries, including South Africa (12). Thus, targeted HIV testing enhances efficient resource allocation and contributes to the efforts aimed at ending the HIV epidemic (13). For instance, multiple machine learning models demonstrated strength in effectively classifying HIV risk among men who have sex with men (MSM) in China (14). The study recommends clinicians use comparable strategies to help priority groups self-monitor their risk for targeted testing, early diagnosis, and reduce HIV transmission (14). Likewise, a supervised machine learning technique with four different models repeatedly achieved high accuracy in predicting HIV testing among South African adults (15). The use of random forest for enabling data-driven decisions, while leveraging digital platforms to enhance HIV testing programs, was emphasized in the study (15).

Furthermore, a key function of machine learning is mitigating the tendency of false HIV test results (5). Elkhadrawi et al. (16) achieved more than 90% accuracy in classifying false and true positive HIV results from clinical data in the United States of America (USA). In South Africa, machine learning algorithms deployed as mobile applications achieved 98% sensitivity and 100% specificity in interpreting HIV rapid test images as true positives/negatives (17). Even though HTS counselors are trained in quality assurance, ensuring HIV testing results are highly accurate, human errors are sometimes inevitable (17, 18). Hence, machine learning plays a critical role in ensuring the accuracy of HIV diagnosis.

Moreover, HIVST was introduced in 2016 in South Africa (19); the same year, HIVST and partner notifications guidelines were released by the World Health Organization (WHO) to address stigma, discrimination, and privacy concerns in screening services (20). Many studies found HIVST acceptable to various sociodemographic groups in South Africa, giving preferences to its convenience and privacy over traditional HIV testing (21–25). However, HIVST alone presents limitations in pre- and post-test counseling and lacks the ability to link users' results to care (26). Thus, digital initiatives, including AI tools, have been introduced to address these challenges (21, 26). Studies show that machine learning, when interacting with mobile devices, can facilitate self-pre- and post-counseling, link HIV results to health facilities, and enhance index testing while maintaining users' privacy and confidentiality (27–30). A pilot study using AI conversational agents in mobile devices exhibited strong capability in aiding HIV counseling, with high acceptability reported among users in low-resource settings (9). Machine learning-driven HVST, particularly when accompanied by voice-overs, has been proven feasible and accepted by high-risk individuals (10). According to Ni et al. (31) Machine learning promotes the distribution of HIVST among KPs in China, increasing the uptake of HIV testing.

Despite the opportunity, research has shown that machine learning is underutilized in HIV testing interventions in South Africa. Additionally, machine learning applications in HIV testing in South Africa, from the stakeholders' point of view, remain unclear. This study explored the perspectives of key stakeholders in Gauteng Province on the status of machine learning in HIV testing in South Africa. Insights from frontline workers such as public health experts, HTS counselors, lab scientists, medical doctors, nurses, and program managers are critical in designing policies to integrate machine learning in testing programs.

## 2 Materials and methods

### 2.1 Study design

An exploratory design was used to explore stakeholders' perspectives on the application of machine learning in HIV testing in South Africa. Empirical evidence highlights expanded HIV testing coverage with machine learning in certain countries; however, developing countries, including South Africa, are left behind (5, 32). To understand the status quo, we employed a qualitative method to obtain in-depth knowledge of relevant stakeholders engaged in HIV testing interventions. The qualitative approach was necessary to understand how far South Africa has gone with the integration of machine learning and what gaps and opportunities exist in the implementation.

### 2.2 Study participants

This study purposefully selected 15 key stakeholders from relevant institutions within Gauteng Province, South Africa. The study participants were healthcare professionals and program managers from government facilities and non-governmental organizations (NGOs) in Johannesburg and Pretoria. The healthcare professionals, such as HTS counselors, medical doctors, a retention counselor, and a nurse, work in two government health facilities. The program managers were from different government agencies and NGOs. The inclusion criteria for this study considered stakeholders involved in HIV testing-related interventions who were aged 18 years and had expressed willingness to participate in the study.

### 2.3 Recruitment and data collection

A purposive sampling technique was used to recruit 15 stakeholders from February to March 2025. The purposive sampling technique ensured that relevant stakeholders in HIV testing-related services and programs with an understanding of machine learning applications were recruited for this study. Machine learning is emerging and less utilized in real testing settings in South Africa, requiring insights from individuals with relevant expertise to communicate comprehensive results. Upon receiving ethical approval from the University of Johannesburg and Johannesburg Health District, relevant facilities and organizations were identified. A total of 10 healthcare professionals were recruited from two health facilities in Johannesburg, and five program managers/leads were from NGOs and HIV implementing organizations in Johannesburg and Pretoria. The facility managers were the point of contact, and they later introduced the researcher to the potential study participants. The healthcare professionals were informed about the study by the managers before being referred to the researcher. The researcher reintroduced the study to the participants and gave them the informed consent and information letters to read. Those who were interested and signed the consent forms participated in the study. The program managers were contacted via email, and those who agreed to participate scheduled an appointment with the researcher.

An individual-based, face-to-face, in-depth interview with a semi-structured guiding questionnaire was used to collect qualitative data (Appendix A). The interview guide consists of two main sections. The first part was about the participants' characteristics. The second section asks questions about the status of machine learning in HIV testing in South Africa. The interviews were recorded with a tape recorder, and additional notes were also jotted down during the interviews. Each interview lasted for about 30–45 minutes. The study completed the interviews after data saturation was reached with 15 stakeholders.

### 2.4 Data analysis

The qualitative data were analyzed using thematic content analysis. The Web version of Microsoft Word 365 was used to transcribe the interview audio. The transcripts were read and reread for familiarity and accuracy while listening to the recordings. Before coding, the transcripts were cleaned, and language editing was done without changing the original meaning of the transcripts. The transcripts and additional notes were uploaded to ATLAS.ti version 23.4.0 for analysis. The software developed codes and linked them to meaningful quotes. The results were presented in themes and sub-themes (Table 1).

**Table 1:** Matrix table with examples of codes.

Themes	Sub-themes/codes	Examples of quotes
1 Awareness Levels	1.1 Limited knowledge among frontline workers	“Not that I know of, because what I know is those conventional testing methods; I haven't seen machine learning yet.” (P05)
	1.2 Research vs. implementation gap	“Research has always been done, but the only gap that we have is the communication of research findings to the decision makers and programmers.” (P12)
	1.3 Need for education	” Exactly. Those uneducated people, there are people from rural areas. They never go to school, you understand? So how, then, is it going to be helpful to them?
2 Existing applications	2.1 Self-testing support	“I think it's to be more conducive and more helping regarding those people who are maybe scared to come to the clinic, maybe because of the stigma.” (P09)
	2.2 Data analysis tools	“Would also be able to identify the high-risk individuals. I mean, I think that's something that we all are always looking forward to in ensuring that we're able to give people appropriate services, especially people who are at high risk of acquiring this HIV and not being aware that they are at high risk. So with machine learning, we would be able to do all these.” (P13)
	2.3 Counseling aids	"Young people, especially those who are at higher risk and would need the information seem to be more much more comfortable and engaging with AI and feel less judged than when opening up to an adult service provider.” (P13)
3 Perceived potential	3.1 Youth engagement	“Yeah, it may just be one of those things that will encourage especially the young people to test. They are more techno-savvy.” (P12)
	3.2 System efficiency	“It will reduce the workload for us and it will help us to know if the patient indeed tested, and what the results are I think it will help a lot.” (P01)
	3.3 Data-driven decisions	“It will also improve by correctly identifying those people that are due or that need to be tested for HIV. Instead of wasting resources testing everyone, we can focus efforts where they're needed most.” (P11)

### 2.5 Rigor and trustworthiness

The rigor was ensured through the Guba and Lincoln principles of credibility, dependability, confirmability, and transferability (33). (i) Credibility: The researchers (MJ and ED) independently analyzed the data, and an external coder was used to confirm the conceptualization and modifications of the codes. Potential bias due to researchers' expertise and interest in machine learning was noted, and objective model evaluation, with cross-validation, and independent review of research were employed to ensure credibility and mitigate bias. (ii) Dependability: This principle was ensured by coding and recording the transcripts by the authors (MJ and EP) and an independent coder until consistent results were achieved. (iii) Confirmability: The constructions of the themes and sub-themes, as well as the accuracy and alignment of the results, were confirmed by all the co-authors. (iv) Transferability: This principle was attained by providing a comprehensive methodology for replicability. During the recruitment process, the research considered diversity in terms of age, sex, professional roles, and stakeholders' experience.

### 2.6 Ethical considerations

This study was conducted in accordance with the Helsinki Declaration. Before the commencement of the study, the protocol was approved by the University of Johannesburg Research and Ethics Committee (REC-2725-2024). Additional approval was obtained from the Johannesburg Health District (NHRD REF. NO.: GP_202404_085). The researcher adhered to the guidelines of the Personal Information Protection Act (POPIA), and standard ethical principles were followed. The principles of informed consent, voluntary participation, privacy, confidentiality, beneficence, and non-maleficence were firmly guarded. The researcher ensured that participants read and understood the personal information letters, and consent forms were signed before the commencement of the interviews. After the participants signed the consent forms, they were kept in a different file to prevent re-identification. Participants' sociodemographic information and additional notes were written on pieces of paper labeled with unique codes, and the data were anonymized. The recordings strictly contain only conversations around the status of machine learning and HIV testing, and no personal or sensitive topic was discussed. Only participants who agreed to participate voluntarily were included, and they were given the opportunity to withdraw at any stage of the study. The study did not subject participants to any physical or psychological harm.

## 3 Results

### 3.1 Participants' characteristics

The demographic characteristics of the stakeholders are summarized in Table 2. The study included 15 stakeholders, with the majority (*n* = 10) being health professionals from government health facilities in Johannesburg, consisting of HTS counselors (*n* = 6), medical doctors (*n* = 2), retention counselors (*n* = 1), and professional nurses (*n* = 1). Five participants were program managers/leads from both government institutions and NGOs within Johannesburg and Pretoria. Most of the stakeholders were females (*n* = 10) between the ages of 32 and 52 years, with 4–30 years of professional experience in HIV testing-related services.

**Table 2:** Participants characteristics.

Participants’ characteristics	Category	Frequency (*n*)
Age group in years	32–40	8
	41–52	7
Sex	Male	5
	Female	10
Highest qualification	Matric (High School)	2
	Post-Secondary Certificate/ Diploma	5
	BSc/MBBS	4
	MSc/PhD	4
Type of organization	Government Health Facility	10
	Government Organization	2
	Non-Government Organization	3
Organization's location	Johannesburg	12
	Pretoria	3
Profession	HTS Counselor	6
	Retention Counselor	1
	Professional Nurse	1
	Medical Doctor	2
	Program Manager/Lead	5
Years of experience	<5	1
	6–15	12
	16–30	2

HTS, HIV testing services.

### 3.2 Themes and sub-themes on the status of machine learning in HIV testing

**Figure 1:**
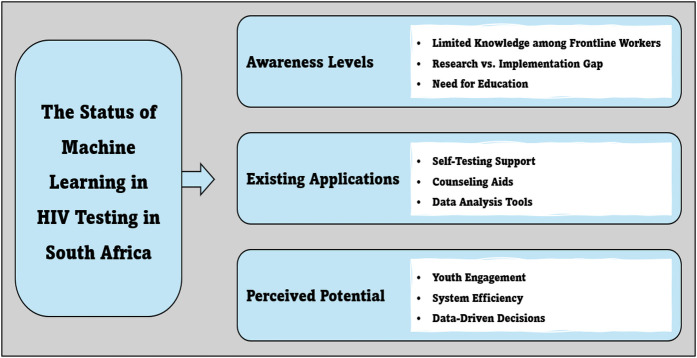
Themes and sub-themes on the status of machine learning in HIV testing in South Africa.

The study produced three main themes describing the status quo on the application of machine learning in HIV testing interventions in South Africa. The central themes and sub-themes shown in Figure 1 are discussed below.

### 3.3 Theme 1: awareness levels of existing machine learning interventions

**Figure 2:**
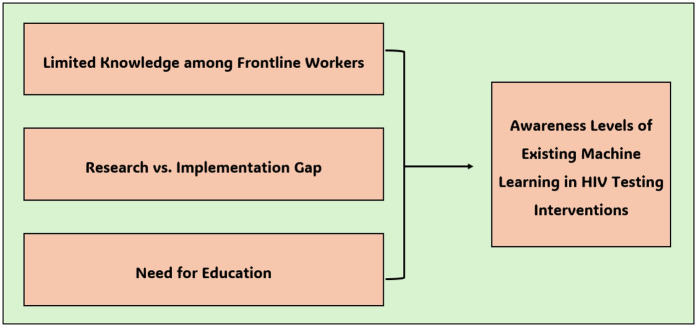
Awareness levels of existing machine learning interventions.

The awareness level of the existing machine learning interventions implemented to enhance HIV testing in South Africa became a central topic of discussion in this study. While awareness levels of such innovations are low, some stakeholders perceive that the benefits of machine learning could be realized if the existing gaps are effectively addressed. This theme was further divided into three sub-themes as illustrated in Figure 2.

#### 3.3.1 Sub-theme 1.1: limited knowledge among frontline workers

The study shows that most frontline workers, especially healthcare workers, have limited knowledge about machine learning and its intersection with HIV testing. When participants were asked if machine learning has been integrated into HIV testing interventions in South Africa, some of them responded,

“No, I don't see them.” (P01, 50 years)

“No, I haven't heard of it.” (P02, 32 years)

“I'm not sure about it. No. Maybe we can see after they implement it.” (P04, 37 years)

“Not that I know of, because what I know is those conventional testing methods, I haven't seen machine learning yet..” (P05, 32 years)

“Currently, I don't think so.. I don't know of any actual programs..” (P06, 32 years).

#### 3.3.2 Sub-theme 1.2: research vs. implementation gap

Even though machine learning is underutilized in HIV testing programs in South Africa (5), stakeholders believe it is important to generate research evidence confirming the effectiveness of machine learning interventions. While this is true, participants expressed a gap in implementing research findings in South Africa. A stakeholder said,

“Research has always been done, but the only gap that we have is the communication of research findings to the decision-makers and programmers. Here's why you will end up with these studies that speak to machine learning and all the goodness that they have. But eventually the same studies are shelved somewhere. If we only focus on research, then most of these findings will remain the research findings, and they will never see daylight in terms of implementation.” (P12, 42 years)

Other participants expressed a lack of effective implementation of research findings in their day-to-day activities,

“I find it truly not much helpful to do research, and it ends at the point where you've done research and you've published, and that's it. But whatever strategy and whatever it is that you are researching, we don't see how we can replicate it and implement it to benefit the broader society.” (P13, 52 years)

“Maybe they have it in the department, from the statistical point of view, but from the day-to-day accessibility, we don't.” (P06, 32 years)

#### 3.3.3 Sub-theme 1.3: need for education

The study shows a need to strengthen education programs on machine learning interventions. For instance, studies reveal that machine learning enhances HIVST through mobile apps. However, some stakeholders claimed that a lack of knowledge of operating such technologies would be a barrier.

“Exactly. Those uneducated people are people from rural areas. They have never been to school, you understand? So how, then, is it going to be helpful to them? That's why I always say that you know what? When the government creates things, think about the civilization of our country. You understand… because people from rural areas…” (P03, 44 years)

“They wouldn't even be aware of machine learning. What is it? So, that lack of knowledge and understanding of the platform itself may just be the barrier because you still have to teach everyone what it is. What is it used for, and how is it used and how will it benefit and also the cost-effectiveness of such platform?” (P12, 42 years)

“So, I would say learning from people who are already using it. And just how to adapt and adopt some of these methods would benefit.” (P13, 52 years)

### 3.4 Theme 2: existing applications

**Figure 3:**
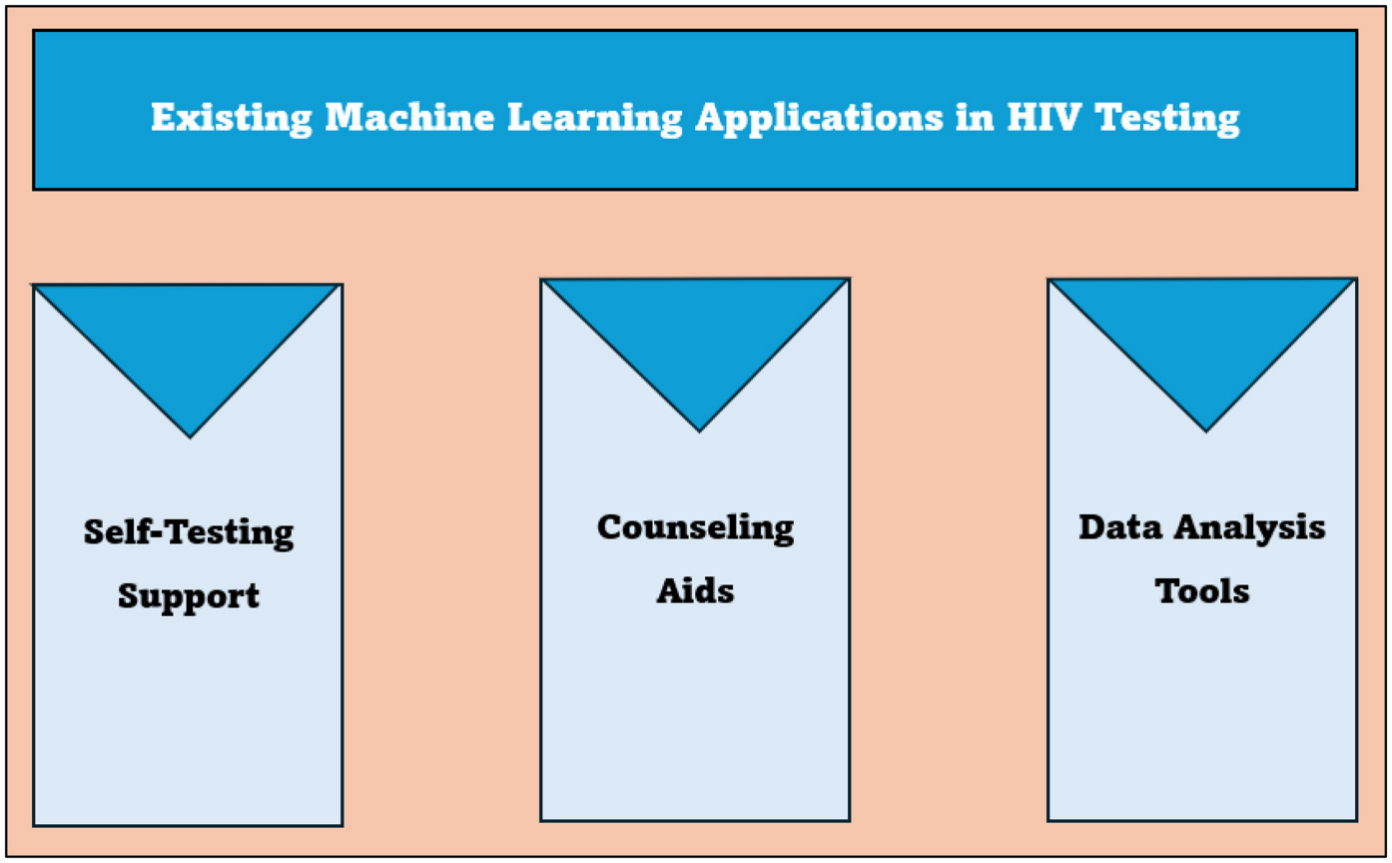
Existing machine learning applications in HIV testing.

The study participants noted existing machine learning applications, such as HIVST support, counseling aid, and data analysis, that are enhancing HIV testing. The applications are further described in Figure 3 below.

#### 3.4.1 Sub-theme 2.1: self-testing support

Recently, studies have integrated machine learning features in mobile applications to aid HIV self-counseling and testing. Some of the stakeholders believe this will make HIV testing more convenient and efficient for both clients and testers. For instance, a participant asserted that machine learning, through HIVST, could address stigma related to HIV testing, especially in a generalized setting.

“I think it will be more conducive and helpful regarding those people who are maybe scared to come to the clinic, maybe because of the stigma.” (P09, 37 years)

A program manager suggested that users would like to provide honest responses to the machine learning-added HIVST tool rather than to the HTS counselor.

“I think it's going to be a great tool, firstly in the areas where we can get to identify people that are eligible for testing… because one of the gaps that we've seen is that in as much as we do have the screening tool that has questions where a tester can actually ask different questions, then people tend to lie there.” (P11, 43 years)

Despite the perceived benefits of HIVST, an HTS counselor claimed a lack of linkage between the existing HIVST devices and health facilities, suggesting the need to address such a gap using machine learning.

“We used to give them HIVST kits to take home but.. we don't get feedback from them.” (P01, 50 years)

#### 3.4.2 Sub-theme 2.2: counseling aids

The study reveals that machine learning or AI can provide clients with information about HIV before taking a test. A stakeholder perceived that machine learning-aided HIV self-counseling and testing would be more appealing to young people who frequently face HIV-related stigma.

“Young people, especially those who are at higher risk and would need the information, seem to be much more comfortable and engaging with AI and feel less judged than when opening up to an adult service provider.” (P13, 52 years)

A tester indicated that clients informed about HIV by machine learning make her job easy.

“Maybe a patient will come with, you know, being informative already. So, my job will just be to conduct the test.” (P02, 32 years)

#### 3.4.3 Sub-theme 2.3: data analysis tools

A key strength of machine learning is its ability to make accurate predictions from complex datasets. Studies utilized these techniques to predict individuals who are most eligible for HIV testing to enhance targeted HIV testing.

“Would also be able to identify the high-risk individuals. I mean, I think that's something that we all are always looking forward to in ensuring that we're able to give people appropriate services, especially people who are at high risk of acquiring this HIV and not being aware that they are at high risk. So, with machine learning, we would be able to do all these.” (P13, 52 years)

“I think maybe your machine learning tool will also be aligned with it…. we also tried to use the index data that we're having because when we are offering index patients, then we collect like the addresses and whatever and we try to map in the communities using some geospatial mapping of some sort to say in which areas.” (P15, 35 years)

“Maybe it will help us reduce the paperwork. Maybe we'll get some new information.” (P04, 37 years)

### 3.5 Theme 3: perceived potential

**Figure 4:**
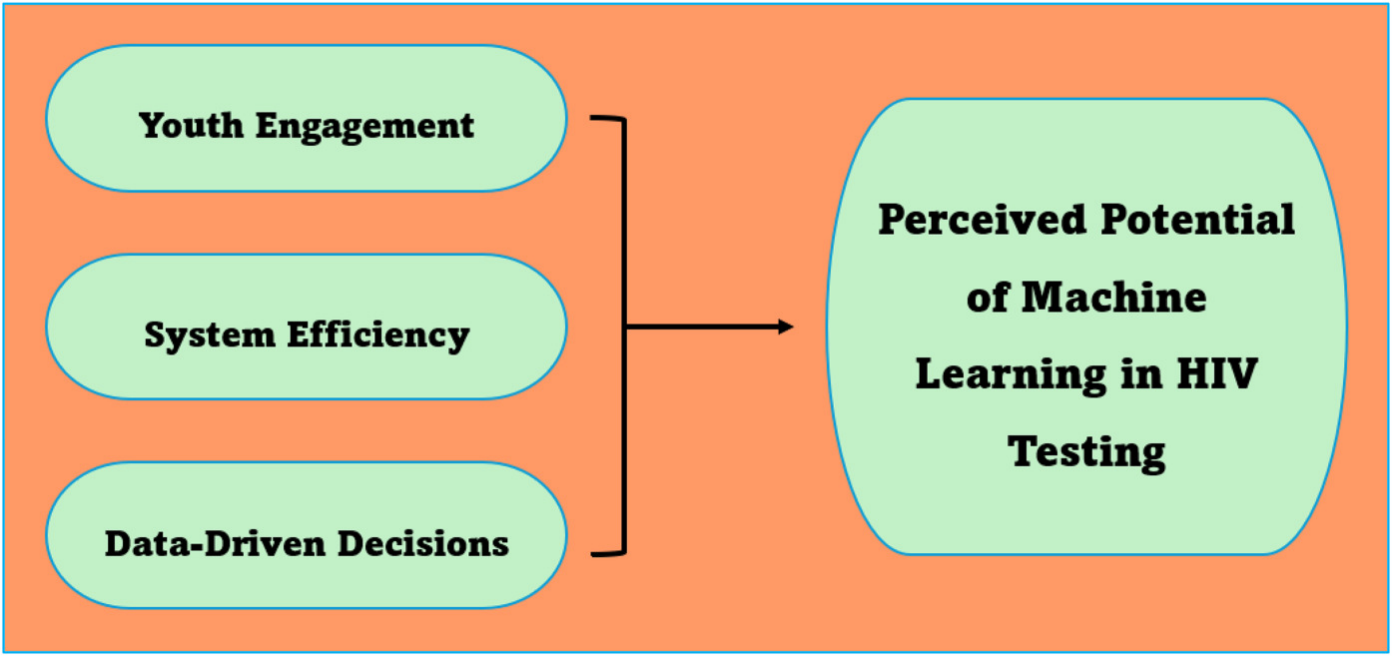
Perceived potential of machine learning in HIV testing.

Machine learning is perceived to be a viable tool for improving HIV testing among youth as well as enhancing data-driven decisions. The perceived potentials of machine learning are elaborated below (see Figure 4).

#### 3.5.1 Sub-theme 3.1: youth engagement

The study reveals that machine learning possesses great potential for increasing the uptake of HIV testing among South African youths. Technology-driven initiatives can increase access to information regarding HIV and testing locations and even assist with pre-and post-counseling in the case of HIVST. Since young people nowadays are technologically inclined, it is perceived that such interventions would be beneficial to them.

“I think because the generation that we are dealing with now are very mobile, they are tech savvy, so anything that has got to do with information technology, AI, machine learning, I think it also assists. For instance, if it's a sporting event, if you put it into technology, then they will come. If it's an HIV-related system, then if it's technology-based, then they will come. So, machine learning will come in handy and assist greatly amongst South Africans in this regard.” (P11, 43 years)

“Yeah, it may just be one of those things that will encourage young people, especially, to test. They are more techno-savvy. So having such platforms that will allow them to interact with someone without really having to present themselves to the facility may just increase our uptake of HIV testing.” (P12, 42 years)

A stakeholder added that,

“Most of the organizations that are using it are using it targeting adolescents and young people because we are all aware that is very wary of getting the traditional counseling from the healthcare providers. So, they would prefer to engage with technology, something online, and say, hey, I want to; I think I've just exposed myself to HIV. What can I do?” (P13, 52 years)

#### 3.5.2 Sub-theme 3.2: system efficiency

Researchers have used machine learning models to predict individuals at high risk of HIV from various data sources. Such predictions ensure optimal use of resources, reduce the workload on healthcare providers and improve HIV testing outcomes. Stakeholders stated the following:

“So, the advantages of machine learning.. I think it would have a higher advantage because.. we are struggling to actually identify the high at-risk population.. AI can actually come in and assist.” (P14, 40 years)

“I think it's going to be quite helpful. If it is able to predict high-risk patients, then it actually makes our job a bit easier.” (P05, 32 years)

“ It will reduce the workload for us, and it will help us to know if the patient indeed tested, and what the results are I think it will help a lot.” (P01, 50 years)

#### 3.5.3 Sub-theme 3.3: data-driven decisions

Predictive models are particularly helpful in designing and implementing data-driven decisions for prioritized HIV testing and resource optimization. Program managers highlighted that,

“You kind of need to come up with a predictive model.. different data sources.. to assist with HIV testing and identification of people at high risk.” (P12, 42 years)

“It will also improve by correctly identifying those people that are due or that need to be tested for HIV. Instead of wasting resources testing everyone, we can focus efforts where they're needed most.” (P11, 43 years)

## 4 Discussion

This study aimed to understand the status of machine learning applications in HIV testing interventions in South Africa by exploring stakeholders' perspectives within Gauteng Province. The study reveals a low utilization of machine learning initiatives in HTS, compounded by gaps in implementing research findings and a lack of awareness among frontline workers and users. Despite these challenges, the findings highlight the potential of machine learning to improve HIV testing. Most stakeholders perceived that machine learning could address barriers to generalized testing, including stigma, discrimination, and privacy issues. A key theme from the study was integrating machine learning into digital devices to facilitate self pre- and post-counseling in HIVST. This innovation is a practical solution to testing gaps in today's world, especially for technology-oriented young people and KPs.

In this study, stakeholders claimed that machine learning is a viable tool for predicting high-risk people for HIV testing. This agrees with studies where machine learning was used in identifying similar groups of people (11, 34, 35). Predictive models in HIV testing ensure that PLHIV who are unaware of their status and their contacts are identified and linked to treatment for reduced HIV transmission (13, 15). South Africa, among many other countries, is facing a deficit in health finances due to the reductions in global HIV funding (12). Hence, the efficient use of national resources for testing programs is imperative. Furthermore, the suitability of machine learning in enabling data-driven decisions was highly praised in this study. Research shows that existing HIV testing predictive models benefit evidence-based decisions, thus improving screening services (3). Thus, policymakers should use predictive models developed by studies to inform HIV testing interventions in South Africa.

Stigma, discrimination, and privacy issues have consistently impeded the uptake of HIV testing worldwide (36–38). Based on our findings, machine learning exerts great potential in addressing these gaps. Most participants believed that using machine learning in HIVST will not only improve self-pre- and post-counseling but also offer the opportunity to feel less judged. Young people are prone to certain risky behaviors, making them vulnerable to HIV, yet they feel less empowered by conventional HIV testing methods. Studies have shown that HIV testing becomes more acceptable to youths when integrated with digital health, including machine learning (26, 39–42). A qualitative study conducted in Cape Town by Janssen et al. (23) shows that HIVST through mobile apps provided guidance and, at the same time, gave users flexibility and freedom to decide who to include during HIV testing. The study further highlighted accessibility and usability issues of smartphones for the older adult and people from rural areas (23). It has been determined in our study that machine learning supports self-led HIV testing programs.

However, the level of awareness regarding machine learning became a critical theme in this study. Healthcare professionals, especially HTS counselors, disclosed a lack of knowledge about any existing machine-learning interventions in routine HTS. This indicates evidence of limited application of machine learning in HIV testing programs besides their statistical functions. Developing countries like South Africa are generally disadvantaged in harnessing emerging technologies due to limited expertise, ineffective infrastructure, and resource constraints. A systematic review by Jaiteh et al. (5) shows low utilization of machine learning in developing countries because of the same factors discussed in this study. To address this challenge, there is a need to provide educational programs on the existing machine learning applications among key stakeholders (43, 44).

Studies assessed the feasibility of advancing HIV self-counseling and testing through machine and digital devices (10, 26, 27, 42). This is particularly important for KPs and young people who mostly avoid generalized HIV testing due to stigma and privacy matters (10). While the existing HIVST services are well-received, concerns about their overall effectiveness have been raised (45, 46). In our study, stakeholders expressed worries about the lack of linkage between HIVST results and HIV facilities. They also noted the issues surrounding access and usability for illiterates, older people, and rural users. With effective educational programs, the participants affirmed that the integration of machine learning could enhance counseling services and improve linkage to HIV care. These findings align with several studies that highlighted similar challenges but ascertained the feasibility of HIVST through digital interventions (10, 23, 27, 45–47).

The study noted implementation gaps in research findings regarding the use of machine learning in HIV testing. Previous studies revealed many advantages of machine learning in terms of improving prediction, diagnostic accuracy, and HIV testing uptake (5, 15, 35, 48–50). These studies usually recommend adopting models that possess high accuracy and precision in facilitating such tasks. However, there are several barriers limiting the integration of machine learning. Despite highlighting perceived potentials of machine learning, the study noted a lack of translating research evidence into practice. Beyond a lack of knowledge and limited expertise, machine learning comes with its own challenges (5, 15). Some models are still developing, making them complex, not user-friendly, and difficult to interpret (51). Simpler models that are commonly used and have been validated by experts should guide HIV testing policy decisions, while newer models are being improved. Additionally, the lack of trust and ethical dilemmas surrounding machine learning continue to exist among policymakers and clinicians. The assurance as to how AI tools protect patients' data remains a debate among experts, hence posing misconceptions and underutilization of machine learning in testing programs (51). Policymakers and machine learning experts should strengthen compliance with data protection regulations and data anonymization to enhance data privacy. The practicality of effectively applying machine learning in HIV testing programs requires the appropriate infrastructure to accommodate modern technologies, but low and middle-income countries fail to meet these technological requirements (5). While this issue was faintly discussed in this current study, a follow-up study should explore infrastructural gaps, ethical issues, and other structural barriers to determine the feasibility of applying machine learning in HIV testing in South Africa. The study underscored the need to develop robust predictive models and frameworks to improve the utilization of research findings in the context of machine learning in HIV testing.

### 4.1 Strengths and limitations of the study

To our knowledge, this is the first study to explore the perspective of stakeholders in Gauteng regarding the status of machine learning applications in HIV testing in South Africa. This will serve as a baseline study for future research on the subject. The study interviewed stakeholders from various healthcare disciplines, such as public health experts, laboratory scientists, program managers, HTS counselors, medical doctors, and nurses. The diversity of opinion in this study was critical in ensuring equitable insights on the status of machine learning are established.

However, some limitations were noted in this study. We only interviewed service providers and program managers, which excluded the perspective of the end-users (patients or public) of HIV testing. A follow-up study assessing the feasibility, acceptability, usability, acceptance, and stigma among HIV testing end-users would enhance the practicality of implementing machine learning in testing initiatives. Nevertheless, the status quo about the utilization of machine learning could be better explained by HTS providers and program implementers due to its novelty and technical requirements. Moreover, the study only recruited stakeholders working within Gauteng Province. Thus, the findings do not represent the views of HIV stakeholders in South Africa. Since this qualitative study used purposive sampling, which is limited in data representativeness and generalizability, follow-up studies should apply quantitative triangulation or mixed methods approach with broader and representative sampling methods. Another potential limitation relates to the researchers' position as public health experts with a deep interest in machine learning. This might have led to unintentional bias in interpreting the results in favor of machine learning. We address this by prioritizing the objective model evaluation metrics and findings were independently reviewed by all co-authors and cross-validated with the existing literature to enhance credibility and minimize bias.

## 5 Conclusion

This study explored the perspectives of key stakeholders in Gauteng Province on the status of machine learning in HIV testing in South Africa. The findings highlighted that integrating machine learning initiatives will potentially improve HIV testing in South Africa. Using machine learning to predict high-risk individuals would help to optimize resource allocation and reduce HIV transmission. Additionally, most stakeholders expressed the capabilities of machine learning in addressing barriers to generalized testing, including stigma, discrimination, and privacy issues, through HIVST for young people and KPs. However, limited utilization of machine learning initiatives in HTS beyond statistical analysis was noted in the study. Major barriers include gaps in implementing research findings and a lack of awareness among frontline workers and end-users. Hence, policymakers should design educational programs on existing machine learning applications and how they aid HIV testing interventions. Tailored training programs on HIV testing predictions for researchers across government institutions and NGOs should be established to increase the use of machine learning in statistical analysis. Service providers should be trained and provided with innovative machine models (e.g., diagnostic devices) designed for improving testing services. Community-based awareness campaigns focusing on the use of machine learning – digital devices to facilitate HIVST should target young people and priority groups, especially in remote settings. Evidence from studies using machine learning approaches should also be translated into HTS for improved HIV testing in South Africa.

## Data Availability

The raw data supporting the conclusions of this article will be made available by the authors, without undue reservation.

## Appendix A

**Table A1:** An in-depth interview guide for stakeholders.

Question and probes	Notes/rationale
Background/icebreakers	
I want to begin by asking some questions about you, your job, and what you do from day to day in relation to HIV testing: • Please tell me a bit about yourself: where do you come from? Do you live in this community? • How long have you been working at this health facility/organization, this department, unit? • What specific type of healthcare services do you provide? For example: Collect and test blood samples for HIV, care for HIV patients, etc….	This section is designed to help the respondent feel at ease and to get to know the participant a little better.
Status of machine learning application in HIV testing in South Africa	
Can we talk about the application of machine learning in HIV testing interventions: 1. What do you think about utilizing machine learning in HIV testing? For example: Machine learning is a form of artificial intelligence that can be applied to HIV testing in the following ways: • As advanced statistical tools for data analysis • Predict people at the highest risk of HIV to facilitate targeted testing • Enhance HIV RDT tools and testing devices • Facilitate self-HIV counseling and testing. • Facilitate index testing 2. Do you think machine learning has been greatly applied to HIV testing in South Africa? Please elaborate ……. For example: • To predict people at the highest risk of HIV to facilitate targeted testing • To enhance HIV RDT and testing devices • To facilitate self-counseling and testing (using mobile applications) • To Facilitate index testing 3. Can the integration of machine learning improve HIV testing? Why do you think so? For example: • Targeting and testing people at the highest risk (those who feel stigmatized at generalized HIV testing centers, e.g., key populations). • Enhance testing devices to reduce false positive/negative results • Facilitating self-HIV counseling and testing through mobile phone applications and devices.	To understand the barriers, challenges and possible successes of the application of machine learning in HIV testing.
Wrap up	
4. Finally: • Is there anything else that you would like to discuss today? • Are there any questions that you would like to ask me about anything that we have discussed today?	
